# Efficacy of *Mycobacterium indicus pranii* Immunotherapy as an Adjunct to Chemotherapy for Tuberculosis and Underlying Immune Responses in the Lung

**DOI:** 10.1371/journal.pone.0039215

**Published:** 2012-07-26

**Authors:** Ankan Gupta, Farhan J. Ahmad, Faiz Ahmad, Umesh D. Gupta, Mohan Natarajan, Vishwamohan Katoch, Sangeeta Bhaskar

**Affiliations:** 1 Product Development Cell, National Institute of Immunology, New Delhi, Delhi, India; 2 Faculty of Pharmacy, Jamia Hamdard, New Delhi, Delhi, India; 3 Experimental Animal Facility and Department of Pathology, National JALMA Institute for Leprosy and Other Mycobacterial Diseases, Agra, India; Fundació Institut d'Investigació en Ciències de la Salut Germans Trias i Pujol, Universitat Autònoma de Barcelona, CIBERES, Spain

## Abstract

**Background:**

The 9-month-long chemotherapy of tuberculosis often results in poor compliance and emergence of drug-resistant strains. So, improved therapeutic strategy is urgently needed. Immunotherapy could be beneficial for the effective management of the disease. Previously we showed the protective efficacy of *Mycobacterium indicus pranii* (MIP) when given as prophylactic vaccine in animal models of tuberculosis.

**Methods:**

We sought to investigate whether MIP can be used as an adjunct to the chemotherapy in guinea pig models of tuberculosis. Efficacy of MIP was evaluated when given subcutaneously or by aerosol.

**Results:**

MIP-therapy as an adjunct to the chemotherapy was found to be effective in accelerating bacterial killing and improving organ pathology. MIP-immunotherapy resulted in higher numbers of activated antigen-presenting cells and lymphocytes in the infected lungs and also modulated the granulomatous response. Early increase in protective Th1 immune response was observed in the immunotherapy group. Following subsequent doses of MIP, decrease in the inflammatory response and increase in the immunosuppressive response was observed, which resulted in the improvement of lung pathology.

**Conclusion:**

MIP immunotherapy is a valuable adjunct to chemotherapy for tuberculosis. Aerosol route of immunotherapy can play a crucial role for inducing immediate local immune response in the lung.

## Introduction

The pandemic of tuberculosis (TB) is an international public health tragedy: estimated 9.4 million new cases of TB in 2008. TB is treatable by drugs, and the World Health Organization has promoted the “directly observed therapy” to improve compliance as chemotherapy needs to be taken for 9-month long duration. Patients who default on therapy have severe risk of relapsing and acquiring drug resistance. Hence, multidimensional approach is desired to effectively eradicate the actively multiplying as well as persistent bacteria from the infected lungs.

During the course of mycobacterial infection, the protective Th1 immune response is dominated by immunosuppressive Th2 response which could facilitate the survival and progression of infection. Immunotherapy could be a potential tool in antimycobacterial therapy since it may boost the Th1 immune response in infected patients and might act as force in shifting the immune response from Th2 to Th1 type. This protective immune response can act in synergy to antibacterial chemotherapy. Several studies have been done to evaluate the immunotherapeutic role of *M.vaccae* [1]–[4] and exogenous recombinant cytokines like IL-2 or IFNγ with variable efficacy [5]–[14]. Newly developed subunit and DNA vaccines have shown some beneficial effect if given post challenge [15]–[20]. Thus considering the requirement of an effective and cheap immunotherapy, further research in this area could be valuable.

*Mycobacterium indicus pranii* (MIP) formerly known as *Mycobacterium w,* is a cultivable, non-pathogenic and rapidly growing saprophyte classifiable in Runyons group IV along with other rapid growers like *M. Fortuitum*, *M.smegmatis*, *M. Chelonae* and *M. Vaccae* on the basis of its growth and metabolic properties [21], [22].

MIP shares a numbers of common B and T cell determinants with *Mycobacterium leprae* [23]. Due to this fact, MIP was first evaluated as the candidate leprosy vaccine. Immunotherapy with killed MIP vaccine was successful in patients with borderline-lepromatous (BL) or lepromatous leprosy (LL) [24]–[28]. Patients receiving the vaccine had rapid clinical improvement and were found to significantly reduce the bacterial burden. Recently we have shown in the mice model of tuberculosis that MIP has higher immunogenicity and protective efficacy than BCG when given as prophylactic vaccine by aerosol or parenteral route [29]. There are several reports emphasizing on the aerosol route of delivery of immunogen to induce local lung immune response [30]–[33].

In this study, we evaluated the efficacy of MIP as an adjunct to standard chemotherapy for tuberculosis, when given by aerosol or parenteral route. We monitored the immune cells accumulation in the lung and also analyzed the *in-situ* expression of different cytokine genes to characterize the modulation of immune response with MIP-treatment in *M.tb*-infected guinea pigs. Protection with MIP-treatment was associated with reduced bacterial loads and pulmonary pathology.

## Materials and Methods

### Animals

Female outbred Duncan Hartley guinea pigs (∼300 g in weight) were purchased from the disease-free small animal facility of Chowdhury Charan Singh Agriculture University, Hisar (CCSH), India and held under barrier conditions in biosafety level III animal laboratory where the animals are housed and maintained in agreement with the guidelines of the Institute's Animal Ethics Committee (Approval Number IAEC # 131/05/07).

### Mycobacteria

MIP and H37Rv were grown in Middlebrook 7H9 medium (BD Difco, NJ, USA) with 0.02% glycerol, 0.05% Tween 80 and 10% albumin-dextrose complex enrichment (BD Difco, NJ, USA) in shaker flasks. Bacteria were harvested in the mid-log growth phase by centrifugation at 2,500 g for 15 min. The bacteria were then washed twice using the centrifugal washing method and suspended in saline at the desired concentration. For the killed preparation of MIP, the bacteria were inactivated by autoclaving for 20 min at a pressure of 15 lb/in^2^.

### Infection of animals

Guinea pigs were challenged with a low dose aerosol of *Mycobacterium tuberculosis* H37Rv in a Madison chamber [34], [35] to establish approximately 50 bacilli per lung.

### Chemicals and drugs

Recently published reports show a wide window of dose of different drugs [36]–[39]. We have chosen the combination of isoniazid (INH), rifampicin (RIF), and pyrazinamide (PZA) with the concentration of 10, 12 and 25 mg/kg body weight respectively in a final volume of 500 µl per animal. All drugs were purchased from Sigma (Sigma Aldrich, St Louis, MO, USA). The RIF was dissolved in 100% dimethyl sulfoxide (DMSO) prior to dilution in distilled water (5% DMSO in the final drug solution). Finally the drug suspension was made in the 40% (wt/vol) sucrose solution to increase the palatability.

### Therapy

**Figure 1:**
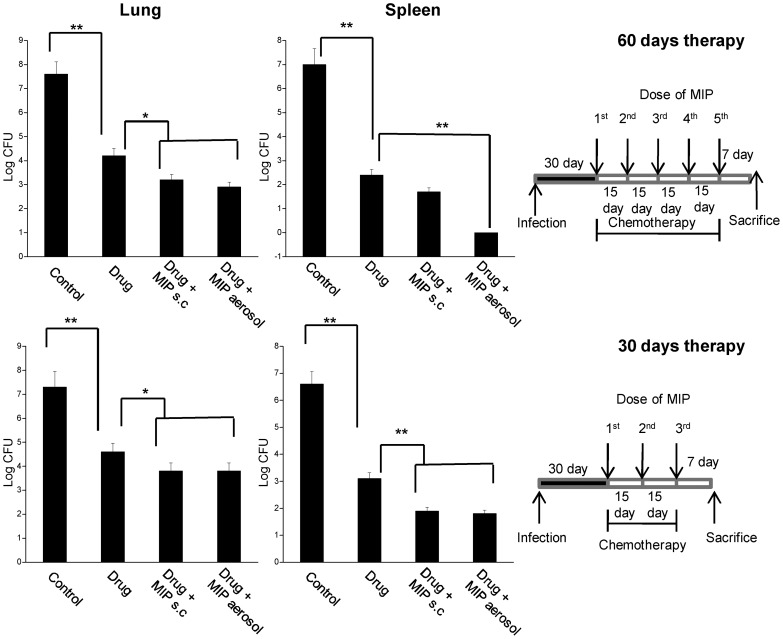
Bacterial load in infected animals following chemotherapy or chemotherapy + immunotherapy. Bacterial load in lung and spleen of guinea pigs following different schedules of therapy as compared to control untreated animals. The chemotherapy was given either for 60 days (1^st^ row) or for 30 days (2^nd^ row). The schematic diagram in each panel shows the detailed schedule of the therapy in the drug-treated and drug + immunotherapy treated groups. Bacterial counts are expressed in log value of colony forming units. Data represents the mean CFU of four animals in each group. * *p*<0.05, ** *p*<0.01.

There were two schedules of therapy as follows:

In the first schedule, the chemotherapy was started from the early chronic phase of infection, i.e., on day 30 post infection and the first dose of MIP was given on the same day. The animals were allocated into the following groups: In the first group only drug combination was given 6 days-a-week through oral route for two months, which accounts for total of 50 doses. The drug was administered orally by force feeding using 1 ml sterile syringe without needle. In the second group, along with the drugs, killed preparation of MIP (10^5^ bacilli in 100 µl saline) was given subcutaneously, every 15 day; which means total of 5 doses of MIP were given at day 1, 15, 30, 45 and 60 respectively during the course of therapy. In the third group, along with the drug combination, aerosol of killed MIP was given (10 ml of 10^9^ bacilli/ml in nebuliser) in a Madison aerosol chamber, every 15 day as described for the second group. As the drug combination was made in the sucrose solution to increase the palatability, the fourth group was sucrose control group, i.e., daily oral dose of 500 µl of 40% (wt/vol) sucrose was given. In the second schedule, the chemotherapy was given for one month, i.e. a total of 25 doses of drugs in the respective groups. The groups which were treated with drug plus immunotherapy, received the dose of MIP by s.c or aerosol route on 1^st^, 15^th^ and 30^th^ day of chemotherapy i.e. total of 3 doses of MIP. The detailed diagrammatic schedule of therapy has been shown in Fig 1.

Briefly, the allocation of the animals in different groups was as follows: 40 animals were taken in one experimental set. 20 animals were taken for each schedule of therapy (so, a total of 20×2 = 40 animals). In each group, 5 animals were taken.

Seven days after the completion of chemotherapy (to allow for clearance of drugs in the animals), the animals were euthanized; organs were aseptically removed for further studies.

### Macroscopic evaluation of lung

After scissoring off the entire lung, the overall pulmonary pathology was graded by giving score to each individual macroscopic parameter as follows: for presence of nodules, scores were 0, 1, 2 and 3 for zero nodule, <5 nodules, 5–10 nodules and >10 nodules respectively. For presence of cavity, scores were 0, 1, 2 and 3 for no cavity, <5 cavities, 5–10 cavities and >10 cavities respectively. For haemorrhagic spots, scores were 0, 1, 2 and 3 for no spot, <5 spots, 5–10 spots and >10 spots respectively.

### Histological monitoring of pulmonary pathology

Lungs from each guinea pig were fixed with 10% buffered formalin. Sections from these tissues were stained using hematoxylin and eosin for monitoring the overall morphology of the section. Progression of lung pathology was evaluated using a histological grading system [35], . The macroscopic and microscopic cumulative score were added to get the gross pathological score of each animal. A pathologist completed the blind scoring of the lung lesions, which was based on randomly selected lung sections from four infected guinea pigs of each experimental group.

### Evaluation of bacterial loads in different organs of animals

Bacterial loads in different groups were evaluated after the completion of therapy. Organ homogenates were plated in serial 10-fold dilutions in quadruplicate on LJ plates (Difco, Beckton Dickinson, NJ, USA). Plates were incubated inside semi-sealed plastic bags at 37°C for 3 to 4 weeks, and colonies were counted. Bacterial loads in 4 individual animals per group were determined at each time point.

### Flow cytometric analysis of cell surface markers

Single cell suspensions were made from the lungs of guinea pigs using a standard protocol [34], [35]. Cells were stained with antibodies against various cell surface markers using the standard methods provided by the manufacturer. Commercially available anti-guinea pig monoclonal antibodies for the following markers were included in the study: CD4 (clone CT7), CD8 (clone CT6), granulocytes (clone MIL4), homing receptor (clone CT4), macrophages (MR-1), MHC class I (clone 2G5) and MHC class II (clone Cl.13.I). All antibodies were purchased from Serotec Inc (AbD Serotec, Oxford, UK). Unfortunately, no other relevant monoclonal antibodies are commercially available for further study. Membrane permeabilization was carried out using Leucoperm (AbD Serotec, Oxford, UK) prior to staining cells with the antibodies for macrophages and MHC class II, as per the instruction manual provided with the antibodies. Samples were run in a FACS Calibur flow cytometer (BD Biosciences), where data acquisition was performed with CellQuest software and analyzed in Win MDI 2.9 software. Analyses were performed on a minimum of 10,000 gated events.

Clone CT4 reacts with a 32–36 kDa glycoprotein expressed on the surface of guinea pig lymphocytes and Langerhans cells. The antigen is anchored to the cell membrane via a phospatidylinositol linkage and mediates T-cell activation. Thus, it may be considered a “guinea pig T-cell activation marker” [41], [42].

### Monitoring of cellular compositions of granuloma

Serial 5–6 µm thick cryo-sections from the lung samples were washed, and non-specific antibody binding was blocked by incubating the sections in a 1% bovine serum albumin-PBS solution. The sections were then incubated overnight at 4°C with primary antibodies, washed 3 times in PBS and incubated with an F (ab') 2 rabbit anti-mouse secondary detection antibody conjugated to horseradish peroxidase (AbD Serotec, Oxford, UK). The color was developed using a DAB substrate kit (BD, NJ, USA) or ImmPACT NovaRED peroxidase substrate kit (Vector Laboratories). The sections were counterstained with methyl green (Vector Laboratories, CA, USA) and mounted with D.P.X. mountant (Fisher Scientific, MA, USA).

### Evaluation of different cytokine gene expression

The mRNA expression levels of different cytokines were carried out by RT-PCR analysis using the standard protocol. The quantitative analysis of mRNA expression levels of different cytokines was performed using qPCR mix (Hot FIREPol® EvaGreen® from Solis BioDyne, Tartu, Estonia) and the samples were run in mastercycler realplex^4^ (Eppendorf, Hamburg, Germany). Primer sequences for IFNγ [43], TNFα [43], IL 10 [44], IL-12p40 [45], TGFβ [46] and CCL5 [47] were taken from respective previously published reports. Primers for IL-2 and GAPDH gene were designed in the software Beacon Designer 7.7 (Premier Biosoft) and the sequences are as follows:

**IL-2**:

Forward: **5**′ GCTACTCTTGTCTTGCCTTG **3**′.

Reverse: **5**′ GGTTGCTAGTAACGCCTTC **3**′.

**GAPDH**:

Forward: **5**′ GTGAAGCAGGCATCAGAG **3**′.

Reverse: **5**′ AGCGTCAAAAGTGGAAGAA **3**′.

### Statistical analysis

Results are shown as the mean ± the standard error. Comparisons between groups were performed by analysis of variance in GraphPad InStat software (Version 3.05). *Student's t* test, was used to assess the statistical significance of difference between the groups.

## Results

### Effect of therapy on bacterial loads in different organs

After 60 days of therapy, significant reduction in pulmonary as well as splenic bacterial load was observed in the groups treated with drug plus immunotherapy as compared to the group treated with chemotherapy only. No bacterium was observed in liver in any of the treated groups (data not shown). In short-course therapy of 30 days, bacterial load in lungs was reduced by about 3 log in drug-treated group as compared to the untreated control group. The load was further reduced by 0.7 log in drug + immunotherapy group (Fig 1). Importantly, in the ‘drug only’ group, bacterial load in the lungs was reduced by about 0.4 log between day 30 and day 60 of therapy. Whereas in the drug+MIP group, reduction in bacterial load in lung during the same period was about 1 log.

### Effect of therapy on pathology of lung

The pathology developed in the lung was assessed in the randomly taken tissue sections from all parts of the organ. The degree of granulomatous organization of infiltrated cells indicated the extent of host response against the infection. The pathological deterioration in the infiltrated area was estimated by evaluating the degree of mineralization, caseation and necrosis.

**Figure 2:**
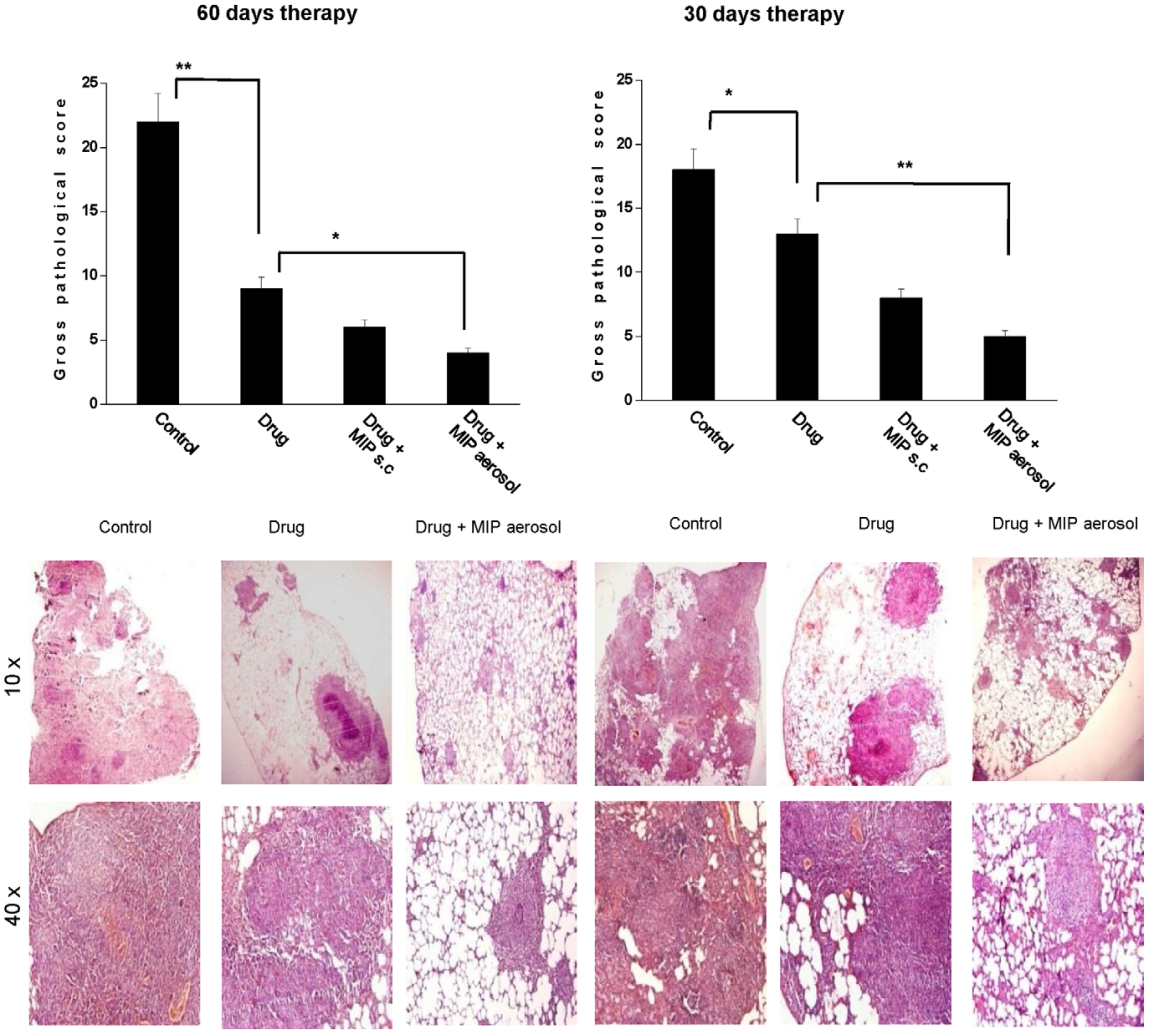
Evaluation of lung pathology. Lung pathology was monitored both microscopically and macroscopically. Bar diagram shows gross pathological score of each experimental group after 60 days or 30 days of therapy. Data represents the mean value of four animals in each group. * *p*<0.05, ** *p*<0.01. Gross pathological score was calculated based on the scoring system which represents number of nodules, haemorrhagic spots, cavities, degree of necrosis, caseation etc as mentioned in the material and methods. Histopathology of lungs of different experimental groups was evaluated after 60 days or 30 days of therapy and the representative photomicrographs are shown below the respective graphs., The microscopic sections from control group, group treated with only drug and drug + MIP aerosol group have been shown in the upper row (10×) and in the lower row (40×). Photomicrographs of lower magnification show the overall distribution of granuloma with plenty of healthy alveolar spaces in drug+MIP group; while the 40× images show the organization of granuloma.

The overall improvement in the pathology of the lung in drug plus MIP group was reflected in the significantly reduced score of gross pathology both after 30 days as well as 60 days therapy (Fig 2). Formation of granuloma in the drug plus immunotherapy group was more localized and the healthy alveolar structure was moderately retained after 30 days therapy. After the completion of 60 days therapy, we found some secondary structures of granuloma in the drug group; whereas in the drug plus MIP group, plenty of primary foci of granuloma were observed. However, in the later group, some diffused nongranulomatous infiltrations were also noticed.

### Kinetics of different immune cells and MHC expression in the infected lung

Since pulmonary pathology and bacterial load was less in the group where MIP therapy was given by aerosol route as compared to subcutaneous route, hence the former group was selected for further studies. To understand the underlying cellular immune response in the group treated with MIP, we analyzed the proportion of different immune cells in the lung at different time points during the course of therapy and compared the same with the group treated with drug only after establishing chronic infection.

**Figure 3:**
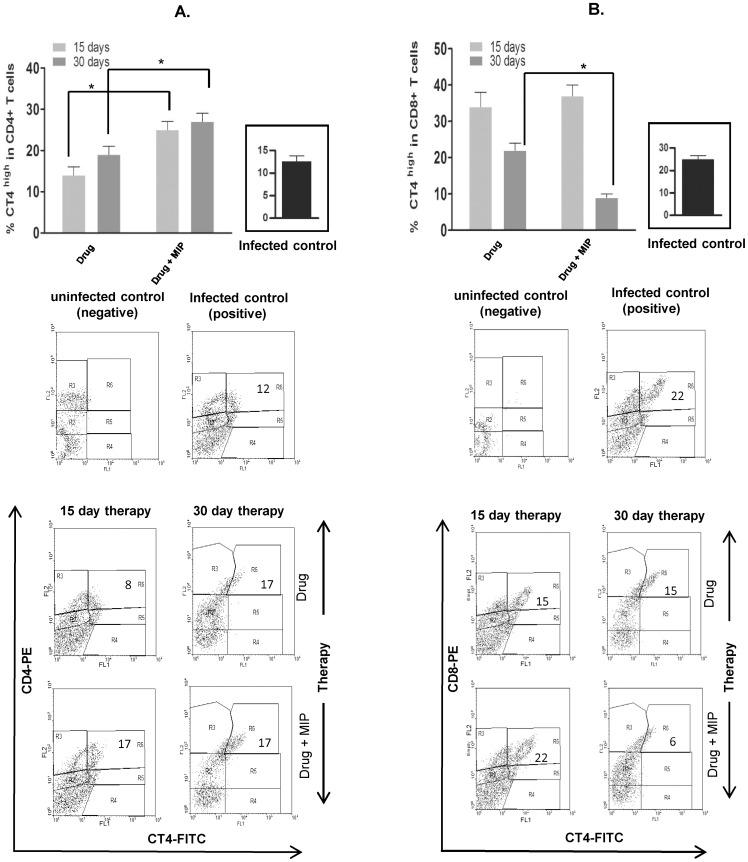
Monitoring of activated T cells in the infected lungs during therapy. Percentage of activated CD4+ T cells (**A**) and CD8+ T cells (**B**) in drug or drug plus MIP group were evaluated at different time points during the course of therapy. The percentage of respective cells in infected control group has been shown in the inset of bar diagram. Activated T cells were analyzed based on the expression of homing receptors as shown in the representative contour plots and compared at 15 days and 30 days of therapy. In each plot, region R6 represents T cells with high homing receptor expression and the corresponding percentage value is mentioned inside the region. Data represents the mean value of four animals in each group. * *p*<0.05. Uninfected animal (naive) was taken as uninfected negative control and untreated animal at 30 days post infection was taken as infected positive control.

Higher proportion of activated CD4+ (Fig 3A) and CD8+ T cells (Fig 3B) was observed after 1^st^ dose of MIP. But the percentage of the activated CD8+ T cells in this group went down at later stage of therapy. Two distinct populations of homing receptor expressing lymphocytes were observed (**representative dot plots in** Fig 3). At this moment, it is hard to interpret this observation but one may speculate that the expression is at ‘high level’ when these are activated maximally to exert biological effect.

**Figure 4:**
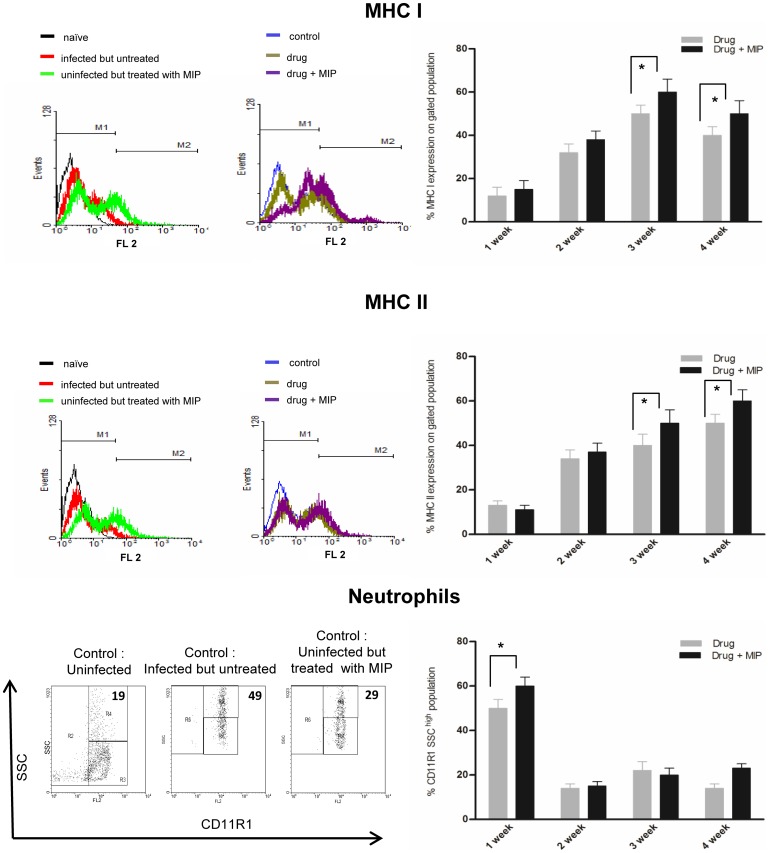
Evaluation of neutrophil infiltration and MHC expressions in the infected lung. The expression of MHC I, MHC II and percentage of neutrophils were monitored at different time points during the course of therapy. The bar diagram shows the mean percentage of MHC expression on gated population or neutrophil content in the lungs of drug or drug plus MIP group. Data represents the mean value of four animals in each group. * *p*<0.05. Entire lymphocyte negative region was gated based on forward versus side scatter and MHC expression or neutrophil infiltration was analyzed on the gated region. The representative plots show the MHC expression or neutrophil infiltration in different experimental groups along with the suitable negative and positive controls. Percentage value has been mentioned in the assigned region in each plot.

We found consistently higher expression of MHC I in the group treated with drug plus immunotherapy as compared to the drug-treated group (Fig 4)**.** Similarly significantly higher expression of MHC II was observed in the drug plus immunotherapy group as compared to the only drug group in the later half of the 30 days therapy (Fig 4).

The percentage of neutrophils was evaluated by gating the granulocytes based on side scatter assuming the higher side scatter of neutrophil population due to high granularity (**representative dot plots,** Fig 4)**.** Interestingly, there was about 10% higher infiltration of neutrophils, 7 days after 1^st^ MIP dose but it came down within a week.

### Effect of therapy on the expression level of various cytokines in the infected lungs

**Figure 5:**
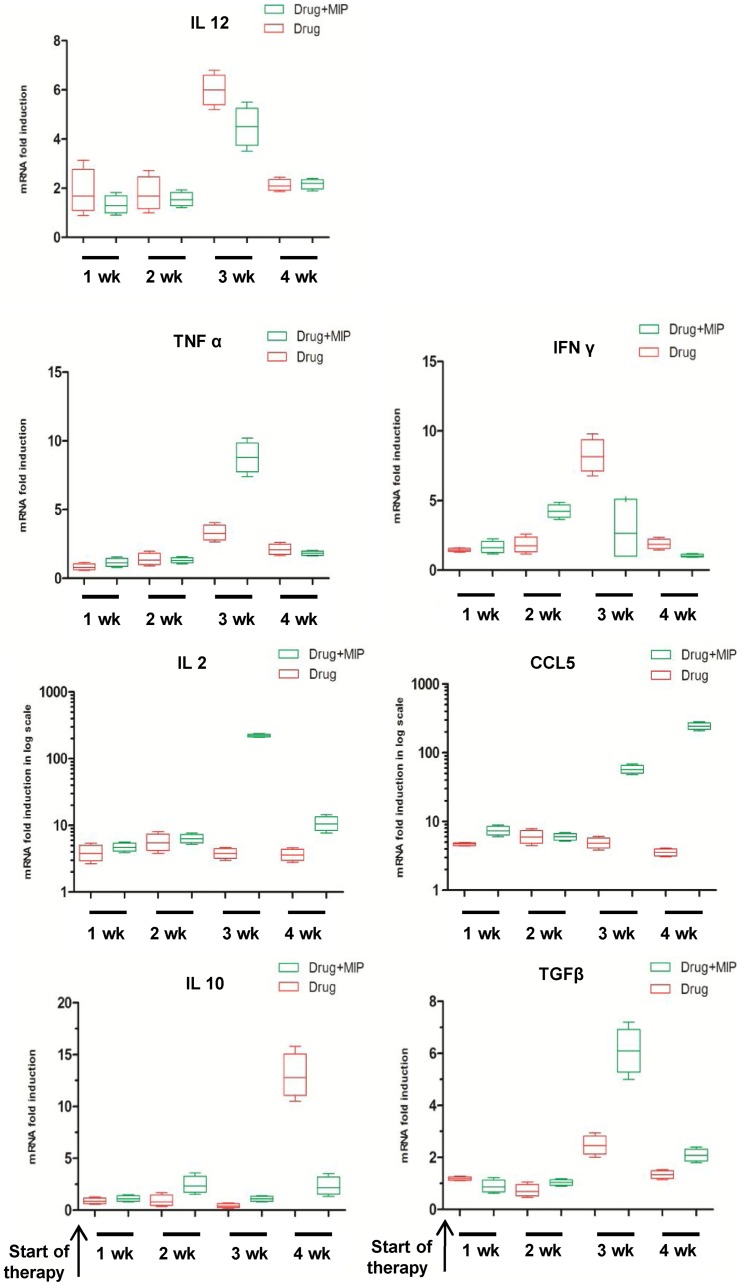
Effect of therapy on mRNA expression level of various cytokines in lung. The expression level of mRNAs for different cytokines were measured by relative quantification method. Expression level of each gene was normalized against the house keeping gene GAPDH and calibrated against the expression of the same gene versus GAPDH in uninfected (naive) animal. Data represents the mean value of four animals in each group.

To further evaluate the local lung immune response modulated by immunotherapy in drug + MIP group, we analyzed the expression of genes for various cytokines (Fig 5). Significantly higher expression of TNFα was observed 7 days after the administration of 2^nd^ dose of MIP. IFNγ expression was up-regulated 15 days after the 1^st^ MIP dose and the mRNA expression level was about 2 fold high as compared to the drug-treated group; however, subsequent to the second dose, the expression level was reduced by 50% in the previous group, which at the same time, spurt up dramatically about 4 times in the drug-treated group. There were no significant differences in the expression levels of TNFα or IFNγ in these two experimental groups after 30 days of chemotherapy. mRNA expression level of IL-2 was increased by more than a log 1 week after 2^nd^ dose of MIP in group receiving immunotherapy; although the level was reduced at subsequent time point, however, it still maintained a significantly higher level of expression as compared to ‘drug only’ group. No significant difference was observed in the expression level of IL 12 between drug or drug plus immunotherapy-treated groups at any phase during the course of therapy, but a radical rise in CCL5 expression was noticed 7 day after 2^nd^ dose of MIP and the high level of expression was maintained till the end of therapy. Initially 2 fold higher expression level of IL 10 was observed 15 days after the administration of 1^st^ dose of MIP in the immunotherapy group as compared to the drug-treated group; the level was somewhat maintained in the drug plus immunotherapy group while the expression level was increased by a log in the ‘drug only’ group at the end of the short-course therapy. The expression level of TGFβ was more than 2 fold high, 7 days after the administration of 2^nd^ dose of MIP in immunotherapy group as compared to the group treated with chemotherapy only; however, the expression level in both groups came down at the subsequent time point.

### Monitoring of cellular composition of granuloma

**Figure 6:**
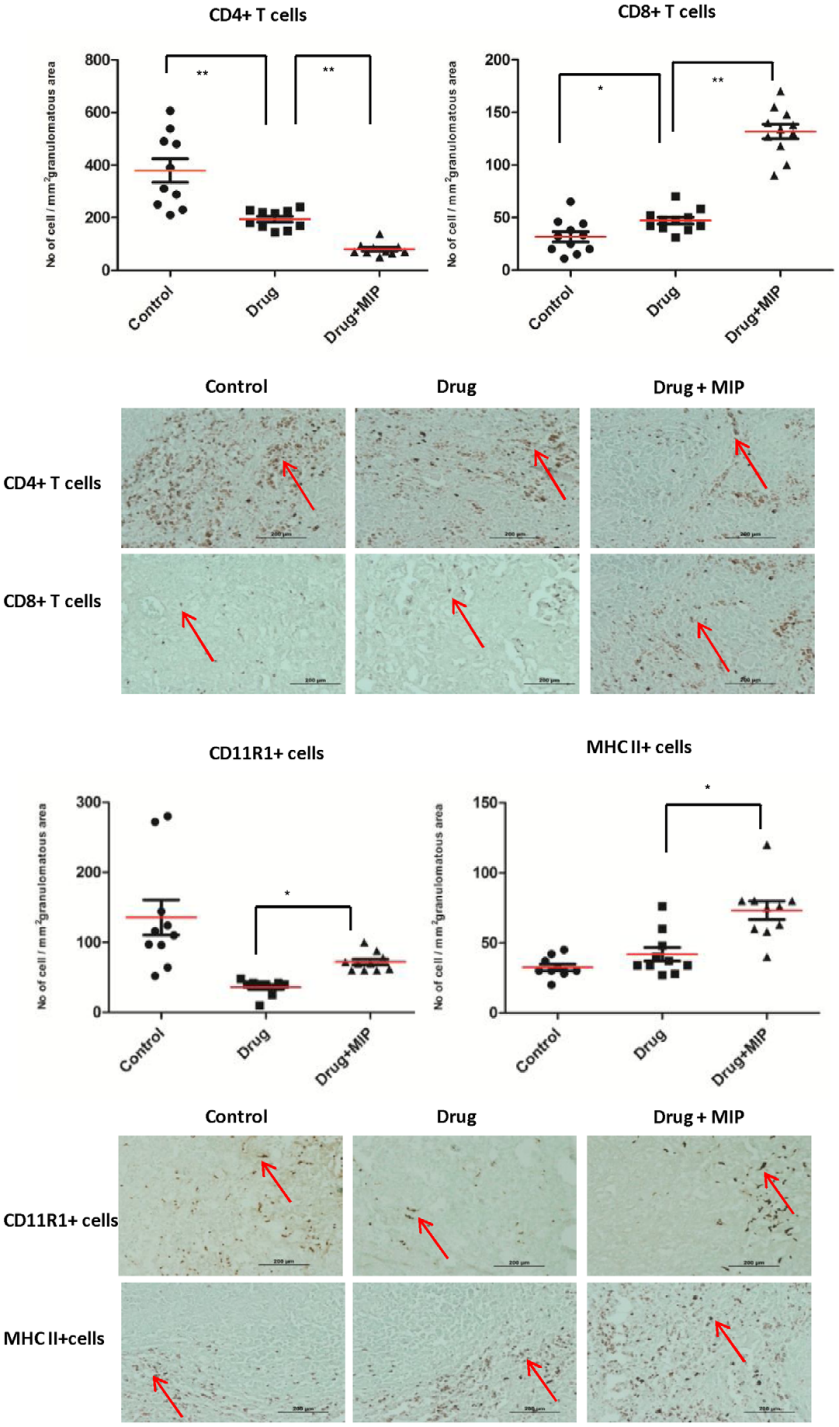
Evaluation of cellular immune response inside the granuloma of infected lungs. Scattergrams show the numbers of CD4+ T cells, CD8+ T cells, granulocytes and MHC II+ cells per unit area of granuloma from different experimental groups after 30 days of therapy. The number of different immune cells inside the granuloma in the lung sections from different experimental groups were counted microscopically and expressed as number of cells per unit area of granuloma. Minimum of 1000 cells were counted from randomly selected, at least 10 fields. Sections were taken from lung samples of 4 animals per group. The representative photomicrographs show the positively stained cells with red arrow in control group, drug or drug + MIP group in each panel. DAB was used as substrate in staining granulocytes while VIP red was substrate for staining other types of cells. * *p*<0.05, ** *p*<0.01.

Formation of granuloma is the hallmark of anti TB host immune response. So we monitored various immune cells inside the granuloma of the infected lungs (Fig 6). Very high proportion of CD4+ T cells was noticed inside the granuloma of the control group. The number of this subpopulation of T cells was 2 fold high in the granuloma of the drug-treated group as compared to the drug+immunotherapy group. The proportion of intragranulomatous CD8+ T cells was about 2.5 fold high in the drug plus MIP treated group as compared to the drug-treated group. Influx of granulocytes per unit area of granuloma in the drug + immunotherapy group was more than 50% higher as compared to the group which received chemotherapy only. In the previous group, we found about 50% higher expression of intragranulomatous MHC II as well. We did not study the cellular composition of granuloma after completion of the 60 days therapy since, the treated lungs had already regained the normal architecture and morphology by this time and granulomatous response was reduced due to reduction of the infection.

## Discussion

Most of the candidates for immunotherapy act through modulation of cellular immune response; Th1 response against *M.tuberculosis* is increased and the immunosuppressive response is down-regulated. We have shown previously that MIP is a potent immunomodulator and generate strong Th1 response. So we sought to investigate its effect when given as an adjunct to standard chemotherapy for tuberculosis.

In this study, we evaluated whether immunotherapy could be beneficial as an adjunct to the standard chemotherapy. We further investigated the possibility of reducing the duration of chemotherapy when given with adjunct immunotherapy. We also studied whether early administration of MIP (before start of chemotherapy) and then followed along with chemotherapy could provide any additional therapeutic advantage; however we did not observe any significant improvement of bacterial clearance or any other advantage as compared to the group where immunotherapy started along with chemotherapy (data not shown).

We used multiple doses of MIP for immunotherapy as pre-existent Th2-driving physiological condition like infection with *M. tuberculosis* might overshadow the efficacy of a single dose of immunomodulator that could otherwise be effective in eliciting strong anti-TB Th1 response. So, multiple doses of Th1 stimulator might be needed at regular interval to avert such Th2 dominating situation. Secondly, with immunotherapy, strong effector immune response is desired to get quick and synergistic bactericidal effect which could potentiate the standard chemotherapy.

We have evaluated the immunotherapeutic activity of MIP when given as aerosol or by s.c route. The aerogenic route of antigen delivery is not only advantageous over parenteral route for physically targeting the lung, but also due to its noninvasive nature. Administration of MIP by aerosol route is crucial in inducing local lung immune response. There are several reports showing the advantage of aerogenic route of immunization over the parenteral route [30]–[33]. We also reported previously that MIP gave higher protection when given as aerosol than by s.c route in mice model of TB [29]. In this study, improved pulmonary pathology was observed after 30 days in the MIP aerosol group as compared to MIP s.c. group although bacterial load was similar. At later time point MIP immunotherapy was found to be more effective in reducing bacterial load and improving pulmonary pathology when given by aerosol than by s.c route.

The maximum bactericidal activity of the drug combination takes place in the first month; thereafter the bacterial killing slows down [38], may be due to the caseation and mineralization of the granuloma, where the residual bacteria survive due to poor penetration of the drug. This might be the reason for more or less similar bacterial load observed after 30 and 60 days of chemotherapy in the ‘drug only’ group. But the group which received adjunct immunotherapy, pulmonary bacterial load was further reduced by approximately 1 log between day 30 and day 60 of therapy, suggesting that the killing of bacteria during this treatment-period is immunotherapy-dependent.

Higher proportion of activated T cells were observed in the lung, 15 days after start of immunotherapy and also intragranulomatous CD8+ T cells were higher in number in the MIP group which suggests that, one of the early bactericidal effects of MIP-driven immunotherapy could be due to cell-mediated cytotoxic killing of mycobacteria. However, it is not very clear why the number of activated CD8+ T cells declined rapidly after the subsequent doses of MIP. This could be due to the reduced lung bacterial load during that time.

About 10% higher influx of neutrophils was observed within a week after first dose of MIP in drug + immunotherapy group; however the level subsequently trimmed down at subsequent time points of therapy. Recent report suggests that neutrophils play important role in early mycobacterial control [48], [49]. It may be assumed that early high influx of neutrophils might be due to the local mucosal response because of aerosol delivery of MIP; which might be contributing towards the protection as aggravated pathology was not observed at any time point. However, it is not known, whether influx of neutrophils has any role in killing the disseminated bacteria as reported elsewhere in the case of pneumococcal infection [50].

It is very difficult to digest the mineralized or caseated tissue portions to have the good yield of cells. Hence, flow cytometric analysis of the cells from whole lung may not truly reflect the actual *in vivo* condition. So the cellular composition inside the granuloma was also studied by immunohistochemistry. Higher percentage of CD4+ T cells were found inside the granuloma of control as well as ‘drug only’ group. The role of this bulk of T cell subpopulation is not known, but one could anticipate that it might contribute towards the formation of large central necrotic core. We found high intragranulomatous CD8+ T cells in the lung sections of animals which were given adjunct immunotherapy. This suggests the cell-mediated cytotoxic killing of intracellular mycobacteria inside the granuloma.

The expression of intragranulomatous MHC II was more fringed in the drug-treated group (data not shown), while the same was very much disbanded throughout the granuloma in the group treated with drug plus immunotherapy, which possibly suggests the induction of a stronger and uniform macrophage activity in this group. The homogeneous expression of MHC II inside the granuloma could be due to the aerosol route of MIP delivery which allows the overall homogenous spread of MIP antigens.

Interestingly, plenty of granulocytes were found inside the granuloma in the drug + immunotherapy group at late stages of therapy; although unlike immunotherapy with BCG, no Koch phenomenon was observed following multiple doses of MIP during therapy. It is reported that antigranuloma therapy in tuberculosis using prednisone or etanercept as an adjunct to the standard chemotherapy could be a potential therapeutic tool [51], [52]. We believe that in the immunotherapy group, after the initial effective granulomatous response, intragranulomatous granulocytes found at the later stage might disrupt the organization of granuloma and thus disseminated bacteria would be available for the bactericidal activity of the drug combination.

TNFα and IL12 expression level was increased after 2 week of therapy in both the treated groups, could be because of active killing of bacteria at this stage. Higher expression of IL-2 after the second dose of MIP might be responsible for increased expression of CCL5. This high expression of CCL5 in immunotherapy group might be enhancing the T cell recruitment at the site of infection. At later stage, when bacterial burden is low, higher expression of TGFβ was observed which might be contributing towards reduced pulmonary pathology in the immunotherapy group. It is not clear, why consistently high expression of CCL5 was observed; however, it is assumed that it might be the recall response due to multiple doses of MIP.

Unlike the BCG-based immunotherapy [53], we did not find any pyogranulomatous response in MIP-treated animals. A recent report showed that repeated exposure to mycobacterial antigens promotes enhanced IL-17 dependent pathological consequences and repeated BCG-vaccination post *M.tb* exposure results in increased level of IL-17 along with influx of granulocytes which collectively results in lung tissue damage and aggravation of pathology [54]. This finding is very crucial in the context of our work as we have also used multiple doses of mycobacteria following *M.tb* challenge but interestingly we did not notice any sign of pathological deterioration although, we noticed increased level of granulocytes inside the granuloma at late stages of MIP-immunotherapy. We could not investigate any influence of IL-17 due to the unavailability of any suitable reagent. It is difficult to conclude the therapeutic superiority of MIP over *M. vaccae*, as the latter one showed significant therapeutic efficacy and enhaced recall IFNγ response in animal models [3], [55]. However, variable efficacy of *M.vaccae* was reported in different clinical trials [2], [4], [56]. There is also report suggesting the therapeutic ineffectivity of *M.vaccae* [57]. Interestingly the immunotherapeutic activity of *M.vaccae* has been shown to be mediated by the strong Th1 immune response viz. IL-2 and IL-12 and the concurrent downregulation of Th2 response [58] which was also observed with MIP immunotherapy in our study. However, a comprehensive clinical trial is required to finally conclude the immunotherapeutic efficacy of MIP.

To summarize, we found an early granuloma-specific immune response in the drug + immunotherapy group, which was followed by granulocyte-driven dismantling of granuloma which might have been useful in killing the bacteria as they become available to the bactericidal activity of the drugs. A balanced inflammatory and suppressive immune response in later part of the drug plus immunotherapy treatment is perhaps useful in the process of restoration of normal tissue architecture and controlling the initial inflammatory reaction. These data provide the first direct evidence of protective efficacy of MIP immunotherapy in tuberculosis and the associated modulation of immune response which can be related to reduction in the lung pathology and control of bacterial growth. The results indicate that MIP might not be able to reduce the treatment duration, but could be potentially very useful in eradicating the persistent bacteria when given with chemotherapy. We believe that the aerosol route of MIP immunotherapy can play a very important role for inducing immediate local immune response in the lung.
